# Uric Acid Is a Mediator of the *Plasmodium falciparum*-Induced Inflammatory Response

**DOI:** 10.1371/journal.pone.0005194

**Published:** 2009-04-17

**Authors:** Jamie Marie Orengo, Aleksandra Leliwa-Sytek, James E. Evans, Barbara Evans, Diana van de Hoef, Marian Nyako, Karen Day, Ana Rodriguez

**Affiliations:** 1 Department of Medical Parasitology, NYU School of Medicine, New York, New York, United States of America; 2 Department of Biochemistry and Molecular Pharmacology, University of Massachusetts Medical School, Worcester, Massachusetts, United States of America; The Rockefeller University, United States of America

## Abstract

**Background:**

Malaria triggers a high inflammatory response in the host that mediates most of the associated pathologies and contributes to death. The identification of pro-inflammatory molecules derived from *Plasmodium* is essential to understand the mechanisms of pathogenesis and to develop targeted interventions. Uric acid derived from hypoxanthine accumulated in infected erythrocytes has been recently proposed as a mediator of inflammation in rodent malaria.

**Methods and Findings:**

We found that human erythrocytes infected with *Plasmodium falciparum* gradually accumulate hypoxanthine in their late stages of development.

To analyze the role of hypoxanthine-derived uric acid induced by *P. falciparum* on the inflammatory cytokine response from human blood mononuclear cells, cultures were treated with allopurinol, to inhibit uric acid formation from hypoxanthine, or with uricase, to degrade uric acid. Both treatments significantly reduce the secretion of TNF, IL-6, IL-1β and IL-10 from human cells.

**Conclusions and Significance:**

Uric acid is a major contributor of the inflammatory response triggered by *P. falciparum* in human peripheral blood mononuclear cells. Since the inflammatory reaction induced by *P. falciparum* is considered a major cause of malaria pathogenesis, identifying the mechanisms used by the parasite to induce the host inflammatory response is essential to develop urgently needed therapies against this disease.

## Introduction

Malaria is one of the more devastating diseases in developing countries with more than one million deaths per year, mostly in children under the age of five [1]. The disease is caused by infection with the *Plasmodium* parasite, being *Plasmodium falciparum* the most deadly parasite species infecting humans. *Plasmodium* replicates within erythrocytes in the blood. The rupture of infected erythrocytes induces a strong inflammatory response in the host that is mediated by factors derived from the parasite. Since most of the pathologies associated with malaria are caused by the excessive inflammatory response triggered in the host [2], identifying pro-inflammatory molecules derived from *P. falciparum* is essential to understand the mechanisms of pathogenesis and to develop targeted interventions.

Two *P. falciparum*-derived molecules that induce inflammatory responses mediated by the activation of Toll-like receptors have been found: glycosylphosphatidylinositol (GPI) anchors [3] and a parasite-induced polymer of degraded heme called hemozoin that is covered with DNA from the parasite [4], [5]. While these molecules may contribute to the malaria-induced pathogenesis, it is clear that other *P. falciparum*-derived inflammatory mediators remain unknown [6]. Recently, uric acid derived from hypoxanthine accumulated by the parasite was described as a source of inflammation in *Plasmodium yoelii*, a mouse malaria model [7].

In this work, we show for the first time that uric acid derived from hypoxanthine accumulated by *Plasmodium falciparum*-infected erythrocytes is a major contributor of the inflammatory response triggered in human peripheral blood mononuclear cells (PBMCs). Interestingly, a clinical trial using an inhibitor of uric acid formation in malaria patients showed a strong reduction of the inflammatory response [8]. Taken together with our observations, these results suggest a major role of uric acid in the inflammatory response to malaria.

## Results

**Figure 1:**
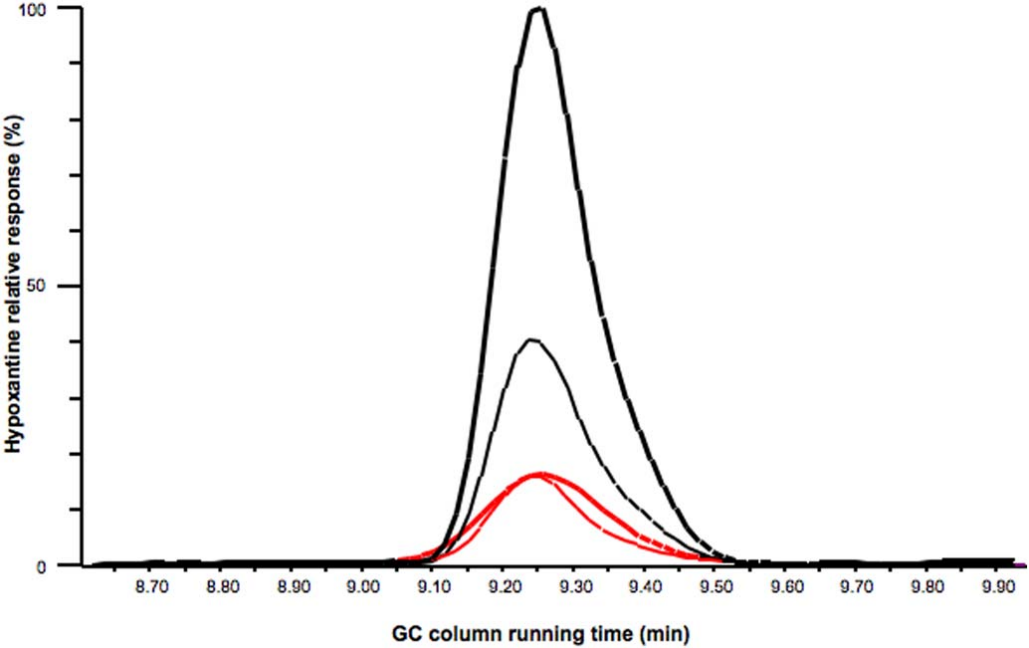
Mature *P. falciparum* infected erythrocytes accumulate high levels of hypoxanthine. Hypoxanthine was analyzed in the soluble fraction of lysates of human erythrocytes infected with *P. falciparum* at different times after infection in a synchronized culture. Lysates of uninfected erythrocytes cultured for the same times were used as controls. Shown are GC - selected reaction monitoring MS ion plots using the m/z 365.2 to m/z 251.2 product ion MS/MS transition. Hypoxanthine accumulated in cultured uninfected erythrocytes (red lines) and *P. falciparum* infected erythrocytes (black lines) is shown. Infected erythrocytes at 33 h (mature trophozoites, thick lines) and 40 h (schizonts, think lines) were purified from synchronized cultures of 2% parasitemia.

Analysis of erythrocytes infected with the rodent parasite *P. yoelii*, showed that they accumulate high concentrations of hypoxanthine and/or xanthine. Enzymatic results using *P. falciparum*-infected erythrocytes suggested that they also accumulate hypoxanthine and/or xanthine [7]. Here, we sought to specifically confirm the accumulation of hypoxanthine in *P. falciparum*-infected erythrocytes and its dependency on the parasite developmental stage. Using gas chromatography (GC) - selected reaction monitoring mass spectroscopy (MS) we determined that lysates of the more mature stages of *P. falciparum*-infected erythrocytes, containing mainly schizonts and late throphozoites, accumulate high levels of hypoxanthine (Fig. 1). This accumulation in the late stages of development is probably the result of a decreased requirement for hypoxanthine by the parasite after the DNA of daughter merozoites has been synthesized. Accumulated hypoxanthine is released by infected erythrocytes after rupture [7], and is converted into uric acid by xanthine oxidoreductase, an enzyme that is present in blood and tissues [9]. Since neither erythrocytes nor *Plasmodium*, have xanthine oxidoreductase activity, the hypoxanthine is not converted into uric acid inside the infected erythrocytes. As expected, the levels of uric acid in lysates of *P. falciparum* schizonts and late throphozoites were very low (below the detection threshold, data not shown).

**Figure 2:**
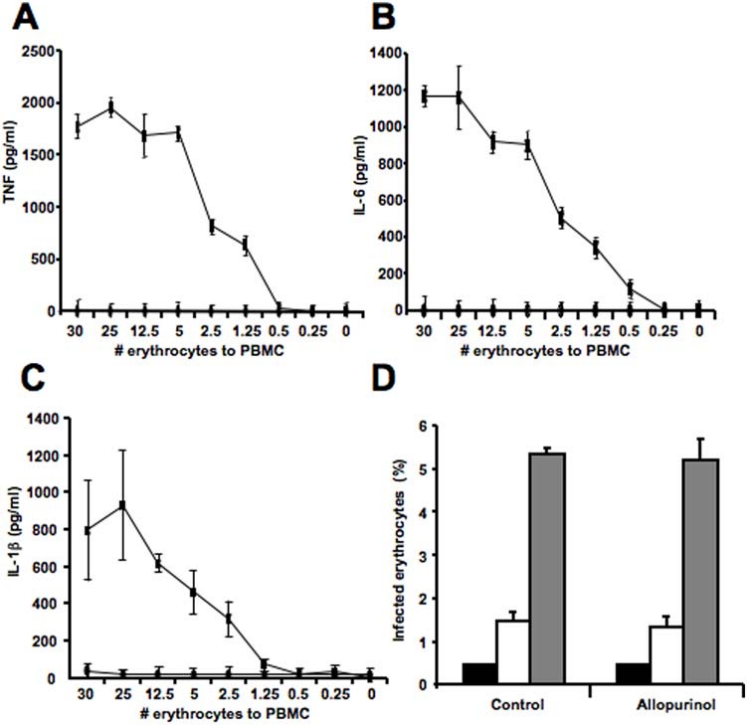
Mature *P. falciparum* infected erythrocytes induce TNF, IL-6 and IL-1β from PBMCs. (A–C) PBMCs were incubated with mature *P. falciparum* infected erythrocytes (squares) or uninfected erythrocytes (circles) at the indicated ratio of erythrocyte to PBMC for 6 h. Data represent the average of triplicated samples with standard deviations. Incubation media were collected and TNF (A), IL-6 (B), or IL-1β (C) concentrations were determined by flow cytometry using cytometric bead array. (D) *P. falciparum* infected erythrocytes were cultivated alone or in the presence of 2 mM allopurinol. Synchronized cultures were seeded at 0.5% rings and the culture media was changed daily. The percentage of infected erythrocytes was calculated after 0 h (black bars), 24 h (white bars) and 48 h (grey bars) of culture.

To study the role of hypoxanthine degradation in the inflammatory response induced by *P. falciparum*, we analyzed the levels of inflammatory cytokines secreted by PBMCs in response to *P. falciparum*-infected erythrocytes [10]. We first performed titration experiments in which PBMCs were incubated with various concentrations of mature *P. falciparum*-infected erythrocytes (schizonts) to establish the optimal ratios of cells. We observed a dose dependent effect of *P. falciparum* infected erythrocytes on PBMC production of the inflammatory cytokines, TNF (Fig. 2A), IL-6 (Fig. 2B) and IL-1β (Fig. 2C).

**Figure 3:**
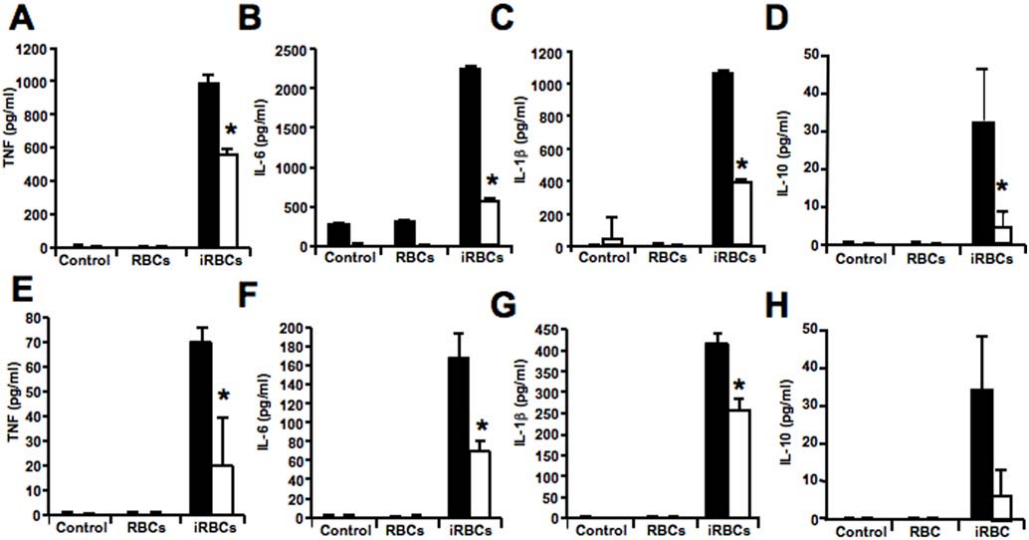
*P. falciparum*-derived uric acid induces TNF, IL-6 and IL-1β release from PBMCs. PBMCs were incubated with media alone (control), uninfected erythrocytes (RBCs) or *P. falciparum*-infected erythrocytes (iRBCs) at a ratio of (5∶1; erythrocyte∶PBMC) for 6 h (24 h for IL-10) in the absence (black bars) or presence (white bars) of 2 mM allopurinol (A–D) or 0.1 mg/ml uricase (E–H). Incubation media were collected and TNF (A,E), IL-6 (B,F), IL-1β (C,G) and IL-10 (D,H) concentrations were determined by flow cytometry using cytometric bead array. Data represent the average of triplicated samples with standard deviations. *, indicates significant differences (*p*<0.05) in cytokine release by PBMCs incubated with iRBCs when compared to IRBCs in the presence of allopurinol or uricase.

To determine if hypoxanthine degradation plays a role in *P. falciparum* induced production of inflammatory mediators, we used allopurinol, an inhibitor of xanthine oxidoreductase that prevents the formation of uric acid from hypoxanthine or xanthine [11]. Despite having toxic effects on other parasites, allopurinol did not inhibit the growth of *P. falciparum in vitro* (Fig. 2D) or *in vivo* [8], [12]. We incubated PBMCs with *P. falciparum*-infected erythrocytes in late developmental stage (schizonts) in the presence or absence of allopurinol to inhibit hypoxanthine degradation. We found that the release of TNF, IL-6, IL-1β and IL-10 by PBMCs was significantly inhibited by allopurinol (Fig. 3A–D), suggesting that hypoxanthine degradation plays an important role in the inflammatory response induced by *P. falciparum*.

To determine if hypoxanthine-derived uric acid was responsible for the inflammatory cytokine response observed, we incubated PBMCs with *P. falciparum* infected erythrocytes in the presence of uricase, an enzyme that specifically degrades uric acid. Similar to allopurinol, we found a significant inhibition of the TNF, IL-6, IL-1β and IL-10 response in PBMCs (Fig. 3E–H). Taken together, these results suggest that uric acid derived from hypoxanthine in *P. falciparum*-infected erythrocytes is a major contributor of the inflammatory cytokine response from PBMCs.

We also measured the levels of uric acid in the culture medium of PBMCs incubated with *P. falciparum* infected erythrocytes, where the inflammatory reaction takes place. We found low levels of uric acid (1.3 µM), which were similar to levels in PBMCs incubated control erythrocytes, and significantly lower than the crystallization threshold of uric acid in biological fluids is (100 µg/ml) [13].

**Figure 4:**
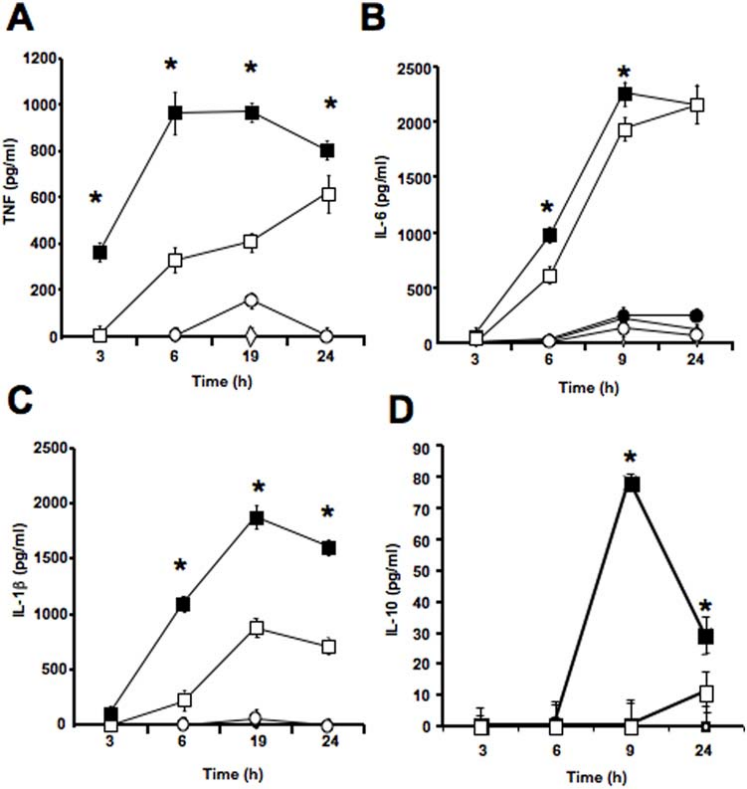
Time course of *P. falciparum*-induced TNF, IL-6 and IL-1β release from PBMCs in the presence of allopurinol. PBMCs were incubated with mature *P. falciparum* infected erythrocytes (squares), uninfected erythrocytes (circles) or media alone (diamonds) at a ratio of (5∶1; erythrocyte∶PBMC) for the indicated time points in the presence (white symbols) or absence (black symbols) of 2 mM allopurinol. Incubation media were collected and TNF (A), IL-6 (B), IL-1β (C) or IL-10 (D) concentrations were determined by ELISA. Data represent the average of triplicated samples with standard deviations. *, indicates significant differences (*p*<0.05) in cytokine release by PBMCs incubated with iRBCs when compared to IRBCs in the presence of allopurinol.

We next characterized the inflammatory cytokine response at different times after incubation of PBMCs with *P. falciparum*-infected erythrocytes. We found that TNF secretion was inhibited at early time points, but was not greatly affected at 24 h (Fig. 4A). These results suggest that the early TNF response is mediated by hypoxanthine degradation into uric acid, but that additional inflammatory parasite-derived molecules trigger the late secretion of TNF. Similar results were found for IL-6, IL-1β and IL-10, although the allopurinol-induced inhibition persisted at 24 h in the case of IL-1β (Fig. 4 B–D).

## Discussion

Since the host inflammatory response contributes decisively to the pathology caused by *Plasmodium* infection [2], finding the parasite mediators that trigger inflammation is essential to design new therapeutic strategies that could interfere with the pathological syndromes induced by the disease. Two different parasite derived molecules, GPI [3] and hemozoin bound to parasite DNA [4], [5], induce inflammatory cytokine responses when purified from parasite lysates. However, the contribution of each to the inflammatory response triggered by the parasite has not been quantified *in vitro* or *in vivo*. Here we quantified the contribution of uric acid in the inflammatory response *in vitro*, showing that uric acid is an important participant, but not the only *P. falciparum*-derived pro-inflammatory molecule activating human PBMCs.

The inflammatory properties of uric acid have been know for decades because of gout, a disease caused by excessively high concentrations of uric acid that crystallizes in the joints triggering an inflammatory response [14]. Uric acid was also characterized as a ‘danger signal’ that is released from necrotic cells and alerts the immune system [15]. More recently it was shown that uric acid is the cause of the adjuvant effect induced by alum [16]. Here we show that hypoxanthine is accumulated in *P. falciparum*-infected erythrocytes and that its degradation into uric acid results in the activation of an inflammatory response from human PBMCs. Our results suggest that malaria, one of the most important diseases throughout human history, may be added to the increasing list of inflammatory processes where uric acid plays an essential role.

We found that uric acid contributes significantly to the secretion of TNF, IL-1β and IL-6 by PBMCs in response to *P. falciparum*-infected erythrocytes. These three cytokines are considered indicative of inflammatory responses, elevated in malaria patients, and associated with the severity of malaria [17]–[19]. TNF has traditionally been used as a reporter to identify the ‘malarial toxin’, a name given to the pro-inflammatory molecules derived from *Plasmodium* [20]. The secretion of TNF by PBMC in response to *P. falciparum* correlates with the stage of the parasite, with schizonts inducing the highest TNF responses and ring stages not inducing any response [10]. Interestingly, we found that the accumulation of hypoxanthine also seems to increase with the progression of the parasite developmental cycle.

We also found increases in IL-10, a well-characterized regulatory cytokine [21], that are dependent on the formation of uric acid. The release of IL-10 after 9 h of incubation is probably a consequence of the initial inflammatory wave of inflammatory cytokines detectable already at 3 h, since inflammatory cytokines such as TNF induce IL-10 release from immune cells [22]. Accordingly, the reduction in IL-10 release after inhibition of the uric acid pathway could be a consequence of the decreased early inflammatory cytokines.

We used both allopurinol, an inhibitor of the generation of uric acid from hypoxanthine and xanthine, and uricase, an enzyme that catalyzes the oxidation of uric acid, to investigate the role of uric acid in *P.falciparum*-induced inflammation. Uricase is a highly conserved ancient enzyme that is found in both prokaryotes and eukaryotes, however, hominoids have lost uricase activity [23]. The lack of an active uricase enzyme in humans is the cause of the high levels of circulating uric acid, which predisposes humans to crystalline deposition and gout [24]. These high levels of circulating uric acid are increased even more during *P. falciparum* infection [25] and can reach levels that are close to the crystallization threshold in biological fluids (100 µg/ml) [13]. Renal dysfunction that is observed during severe malaria infections probably contributes to the increased uric acid levels observed in plasma [26], rendering patients more vulnerable to additional increases of uric acid due to hypoxanthine degradation upon infected erythrocytes rupture. A local increase in uric acid is likely to occur after the rupture of infected erythrocytes, since this is a synchronized event that is perhaps more pronounced in certain tissues where infected erythrocytes tend to accumulate [27]. These high local concentrations of uric acid would lead to inflammatory responses that can be triggered by either soluble or crystallized uric acid [28]. Interestingly, in mice treated with alum, the levels of uric acid were significantly increased [16], but were still three times lower than the urate threshold of crystallization, suggesting that elevated levels of soluble uric acid may have a strong inflammatory activity. We also find uric acid levels below the crystallization threshold *in vitro*, suggesting that soluble uric acid may be the mediator of the response observed in PBMC.

The pathways used by uric acid to trigger inflammatory responses are not fully characterized. It is known that crystallized uric acid induces IL-1β through the NALP3 inflammasome pathway [29], however, the pathway used to induce TNF remains unknown. Using mouse cells, we previously found that the signaling pathway induced by *Plasmodium* is independent of MyD88 (and likely of TLR) and it is different from the pathway used by crystallized uric acid to trigger IL-1β [7].

Interestingly, allopurinol reduces the inflammatory response of humans during malaria. When groups of acute malaria patients infected with *P. falciparum* were treated with allopurinol in addition to the anti-malarial drug quinine, a significantly faster decrease in fever levels and splenomegaly was observed compared to the group of patients treated only with quinine. This reduction of inflammation was observed without a significant decrease in parasite clearance, indicating that the anti-inflammatory effect of allopurinol is mediated by the specific inhibition of an inflammatory pathway and not by a direct effect on the parasite. These results suggest that the hypoxanthine degradation pathway plays an important role in the human inflammatory response to malaria [8].

We have identified *P. falciparum*-derived uric acid as a parasite mediator that triggers inflammation. While treatments to interfere with inflammation-induced pathologies are essential, therapies to reduce the levels of uric acid during malaria infections should not be considered before investigating the effects of uric acid in different aspects of the disease. Since uric acid has also adjuvant and anti-oxidant properties, a decrease may exacerbate certain malaria-associated pathologies.

## Materials and Methods

### Reagents

All chemical reagents were from Sigma unless otherwise specified. Uricase (Elitek, Sanofi-Aventis) was further purified using EndoClean gel filtration columns™ (BioVintage).

### Parasite Culture

Erythrocytic asexual stage cultures of *P. falciparum* strain 3D7 were maintained in culture medium (RPMI 1640, 25 mM HEPES, 10 µg/ml gentamycin, 0.5 mM hypoxanthine, pH 6.75), supplemented with 25 mM sodium bicarbonate and 10% human serum. A 5% hematocrit (O positive, sickle cell negative, leukocyte depleted erythrocytes) was maintained in the atmospheric conditions of 1% oxygen, 5% carbon dioxide and 94% nitrogen. The culture medium was changed daily and the cultures were sub-cultured to maintain a parasitemia of less than 6%. Parasite cultures were kept synchronized using the gelatin flotation method [30]. To determine parasitemia, the number of parasitized erythrocytes from 500 cells in a Giemsa stained this blood smear were counted. Cultures were routinely shown to be free of mycoplasma by PCR.

### Parasite Growth Curve

To synchronize cultures for growth curve experiments, trophozoites were selected by gelatin flotation method [30] 24 h before setting up experiments with ring stage parasites. Triplicate cultures were seeded at 0.5% ring stage parasites in parasite culture media in the presence or absence of 2 mM allopurinol. Cultures were maintained for 48 h without subculturing and the media was changed daily. Parasitemia was determined daily.

### Lysate Preparation

Mature *P. falciparum* infected erythrocytes were purified by the gelatin flotation method [30]. As a control, non-parasitzed erythrocytes were prepared in parallel. 2×10^8^ infected or uninfected erythrocytes were subjected to 20 cycles of freeze thawing. Lysates were then centrifuges at 10,000 g for 10 minutes and the supernatant was collected and dried for GC-MS analysis.

### Quantification of Hypoxanthine by Gas Chromatography – Single Reaction Monitoring Mass Spectrometry (GC-MS)

The dry sample residues were sonicated in 500 µl of HPLC grade water (ThermoFisher), centrifuged and the supernatant removed. Extracts (20% of total) were evaporated to dryness and derivatized to t-butyldimethylsilyl (TBDMS) derivatives and analyzed by capillary gas chromatography – ammonia chemical ionization single reaction monitoring mass spectrometry. 100 µl of this extract was mixed with 200 µl of HPLC grade methanol (ThermoFisher) and the solvent evaporated to dryness under a stream of nitrogen at 60°C. N-Methyl-N-(tert-butyldimethylsilyl)trifluoroacetamide containing 1% tert-butyldimethylchlorosilane (100 µl, Pierce) was added to each sample. The samples were heated at 60° for 30 min to form the TBDMS derivatives. This reaction mixture was analyzed by GC-MS using a Waters Quattro-II triple quadrupole mass spectrometer equipped with an Agilent 6890 gas chromatograph. The sample (2 µl) was injected in the splitless mode onto a 30 m×0.25 mm ID MS DB-5 capillary GC column (J&W Scientific; 1 µm phase thickness) with the injection temperature at 280°C and the column temperature at 210°C. The column temperature was held at 210°C for 1 min, programmed to 260° at 5°/min and held at 260° for 7 min. Ammonia chemical ionization was used with the source pressure at 4e-4 mBar and 175°C. The TBDMS hypoxanthine m/z 365.2 MH^+^ ion was isolated and collisionally activated (25 eV, 7e-4 mBar argon) and its m/z 251.2 product ion (loss of TBDMS) selectively monitored. Peak areas from the selected reaction monitoring ion plots were compared to those from a 3-point hypoxanthine calibration curve from 20 to 300 ng to calculate the amounts of hypoxanthine present in each sample. All analyses were performed in duplicate.

### PBMC Isolation

Leukocyte enriched buffy coats were obtained from the NY Blood Center (Long Island City, NY) and diluted with equal volumes of RPMI (Invitrogen). PBMCs were separated by density gradient centrigugation on 25% Ficoll-Hypaque (GE healthcare). PBMCs were collected, washed two times with RPMI and resuspended in RPMI +10% heat inactivated human AB serum (Valley Biomedicals, Winchester, VA).

### PBMC and Parasite co-culture

Mature *P. falciparum* infected erythrocytes were purified by the gelatin flotation method [30] and incubated with 1×10^6^/ml PBMCs for the indicated time points and ratios at 37°C, 5%CO_2_. The incubation media used was RPMI supplemented with 10% heat inactivated human AB serum, 20 µg/ml gentamicin and 10 mM HEPES. At the indicated time points, incubation media was harvested, centrifuged to remove cells and cytokine were measured. Cytokine levels were measured by ELISA (OPTI-EIA kit, BD Biosciences) or by cytometirc bead array (human inflammatory cytokine kit, BD Biosciences) when indicated according to the manufacturers instructions.

### Statistical analysis

All data were analysed using GraphPad Prism software. Significant differences were determined using one-way ANOVA. Data were considered significant if p<0.05.
